# Hemodynamic and cardiorespiratory ultrasound evaluation by intern

**DOI:** 10.1186/2036-7902-4-S1-A1

**Published:** 2012-12-18

**Authors:** Paula Burgueño, P Garmilla

**Affiliations:** 1 Intensive Care Unit, Hospital Marqués de Valdecilla, Santander, España

## Background

The ultrasound in the ICU has proved to be a non invasive and economic technique that helps the approach in the diagnosis and management of the critical patient. Echocardiography permits diagnosis such as coronary syndrome, pericardial effusion or valvulopathies and brings us the possibility of monitoring the different aspects of shock, like cardiac function or volume respond. Furthermore, lung ultrasound allows us to approach the diagnosis of pneumothorax, pleural effusion, pulmonary edema, consolidation or interstitial disease. For all the abovementioned reasons, we believe intensive residents ought to train in this aspect.

## Objective

To evaluate the resident´s ability to determine the hemodynamic, cardiac and respiratory situation with a basic training in ultrasound.

## Methods

We use VSCAN and lineal transducer probe to do lung ultrasounds in five different areas in each hemithorax. First we examine the parasternal area and then we use the axillar line to divide the lateral of the hemitorax in four parts: anterosuperior, anteroinferior, posteroinferior and postero superior; we are trying to evaluate the possible presence of: pleural sliding, pleural effusion, consolidation, A or B lines, and the correlation with the clinical aspects and X-rays or TC. We use VSCAN for echocardiography and evaluating the cardiac function, to check for the presence of segmentary contractility alterations, valvulopathies and cava vein variability.

We are presented with a 73-year-old patient with previous arterial hypertension, atrial fibrillation, and chronic bronchitis who is admitted in the ICU for septic shock secondary to anastomotic rupture in the postoperative of a colon disease. Thirty-two days later, he is extubated without vasoactive drugs. On the 35th day he started having respiratory problems, fever and hypotension, needing intubation and vasoactive drugs. After a subclavian access, we suspected it to be a left pneumothorax. In the X-rays, both hemithorax bases were observed with an augment of density, mostly in the right lung. A Lung ultrasound was done in the parasternal line of the right lung and we observed pleural sliding with B lines pattern. It was not present in the left lung and we were not able to do the echocardiography because of window absence. TC confirmed the presence of anterior pneumothorax, and a thorax tube was inserted. The clinical situation did not improve. ECG demonstrated a new Q wave in the septal face and negative T in the lateral face. An echocardiography was done and moderate biventricular dysfunction, left ventricle dilated with dyskinetic movement were observed. We also noticed pericardial effusion with a dubious tamponade of the right ventricle, nonetheless this was dismissed because this collapse movement occurred in systole. There were mitral and tricuspid insufficiencies. The cava vein was dilated without variability. At a later time, another lung ultrasound was done where pleural sliding was observed in both hemithorax, with a B line pattern at the parasternal line. Pleural effusion and heterogenic consolidation were noticed at the decline parts of both lungs.

## Discussion

It is a difficult patient with multiple complications that render his hemodynamic situation worse. We were not able to diagnose with certainty the presence of pneumothorax, it is true that M mode could not be done and this had increased the probability of diagnosis of it, but the ultrasound improved the chances and confirmed the drainage of the pneumothorax. We can say that it is a shock with septic and cardiac characteristics with a dilated cava vein and B lines that indicate pulmonary edema. Also bilateral consolidation lung was observed with pleural effusion that makes the pneumonia more likely, bearing in mind the fever and the respiratory problems of the patient.

## Conclusion

Portable ultrasound technology is able to assist physicians in the assessment of the cardiovascular and respiratory system at the bedside of the patient.

Use of the ultrasound can lead to considerable savings of cost and time, as physicians will be able to more selectively order tests based on what is found during the physical examination and after completing a brief ultrasound study. Thus, the ultrasound has the potential to help promote better and more efficient health-care delivery.

The ultrasound is a technology observed-dependent and this is the reason for which a good training is important. More studies are necessary to evaluate the training of the residents on it.

## References

[B1] Hemodynamic monitoring using echocardiography in the critically Springer-Verlag Berlin Heidelberg

[B2] Liechtenstein D Ultrasound diagnosis of the critically ill 2 Berlin-Heidelberg:Springer-Verla 2055

[B3] Colemero M Garcia-Delgado M Navarrete I Lopez-Milena G Utility of the lung ultrasound in the intensive medicine unit Medicina Intensiva 2010 34 9 620 628 10.1016/j.medin.2010.04.004 20483507

[B4] Azcarate JM Ayuela Terre F Clau Ochogavia A Pereira RVicho Role of the echocardiography in the hemodynamic monitorization of critical patients Medicina Intensiva 2011 36 3 220 232 10.1016/j.medin.2011.11.025 22261614

